# COVID-19 Pandemic and Healthcare Workers’ Life and Job Satisfaction: The Role of Stress, Coping, and Self-Efficacy

**DOI:** 10.3390/jcm14248855

**Published:** 2025-12-14

**Authors:** Joanna Dymecka, Jakub Filipkowski, Anna Machnik-Czerwik

**Affiliations:** 1 Department of Health Psychology and Quality of Life, Institute of Psychology, Opole University, Staszica Square 1, 45-015 Opole, Poland; jdymecka@uni.opole.pl; 2 Department of General Psychology and Human Development, Institute of Psychology, Opole University, 45-015 Opole, Poland; jakub.filipkowski@uni.opole.pl

**Keywords:** healthcare workers, job satisfaction, COVID-19 pandemic, stress, coping strategies, social support, self-efficacy, life satisfaction, well-being

## Abstract

**Background:** The COVID-19 pandemic has imposed unprecedented stress on healthcare workers (HCWs), potentially affecting their job satisfaction and life satisfaction. This study aimed to examine the role of perceived stress, self-efficacy, and coping strategies as predictors and mediators of well-being among HCWs during the pandemic. **Methods:** A total of 326 HCWs participated in the study. Perceived stress was assessed using the Perceived Stress Scale (PSS-10), self-efficacy was assessed with the Generalized Self-Efficacy Scale (GSES), life satisfaction was assessed with the Satisfaction with Life Scale (SWLS), job satisfaction was assessed with the Brief Job Satisfaction Scale (BJSS), and coping strategies were assessed with the Mini-COPE. Correlation, regression, and mediation analyses were conducted. **Results:** HCWs reported elevated stress levels (M ≈ 24), higher than general population norms. Stress was negatively associated with life satisfaction and job satisfaction. Self-efficacy and adaptive coping strategies (acceptance, social support) were positively associated with life and job satisfaction and mediated the relationship between stress and life satisfaction. Helplessness mediated the effect of stress on life satisfaction but not job satisfaction. **Conclusions:** High self-efficacy and adaptive coping strategies serve as protective factors for HCWs’ well-being during the COVID-19 pandemic. Interventions enhancing self-efficacy and promoting acceptance and social support may mitigate stress and improve life and job satisfaction.

## 1. Introduction

The COVID-19 pandemic, caused by the SARS-CoV-2 virus, which was first identified at the end of 2019 in China and quickly spread worldwide, has become one of the greatest public health crises of the 21st century [1]. It forced the implementation of extensive preventive measures, such as lockdowns, mobility restrictions, quarantine, and the reorganization of healthcare systems. The pandemic posed challenges not only to the population’s biological safety but also to the economy, education, and social life [2]. One of the groups most exposed to the consequences of the pandemic was healthcare workers [3,4].

HCWs were at increased risk of infection, of transmitting the virus to their relatives, and of the psychological consequences of working under high-stress conditions. Media-reported cases of mortality among medical staff, the high infectivity of the virus, and the initial lack of protective equipment put HCWs in situations of direct threat to their own life and health, as well as that of their close ones [5]. Simultaneously, work overload, due to excessive hours, staff shortages, and the need to care for a large number of critically ill patients, increased the risk of burnout and chronic stress [6,7]. Stressful situations also included difficult ethical decisions—e.g., choosing whom to provide limited resources which caused guilt and moral distress [8]. These challenges could affect healthcare workers’ mental health, increasing stress, perceived threat, and fear and consequently impacting their job satisfaction and quality of life.

Stress can refer to both adaptive (eustress) and maladaptive (distress) responses. In line with Lazarus and Folkman’s transactional theory of stress [9,10], our study focuses specifically on distress—defined as an appraisal in which situational demands are perceived as exceeding available resources and threatening well-being.

A key element of this concept is subjective appraisal—it is not the event itself, but the way it is interpreted that determines the onset of stress. During the COVID-19 pandemic, healthcare workers experienced stress when they subjectively assessed the risk of infection, work overload, and limited protective measures as exceeding their coping capacities [3,11].

Increased perceived stress among HCWs was related to fear of COVID-19, risk perception, and perceived threat [4]. Risk perception is most often associated with perceived severity of illness and perceived susceptibility [12]. Perceived threat refers to an individual’s evaluation of how harmful the consequences of infection would be if it actually occurred. Fear is a characteristic feature of every infectious disease epidemic. During the COVID-19 pandemic, fear arose from the risk of infection, death, and loss of loved ones, as well as contact with potentially infected individuals [2,4]. People were also concerned about serious health complications, forced hospitalization, and long quarantine periods. Another source of fear was the possibility of spreading the infection to relatives who might not survive the disease. Fear of coronavirus in the early months of the pandemic stemmed from its novelty and uncertainty regarding the epidemic’s progression. Therefore, fear of COVID-19 was significantly greater than fear of seasonal influenza [2].

During the COVID-19 pandemic, healthcare workers were exposed to increased levels of stress, anxiety, and perceived threat, which could affect their psychological well-being. Experiencing chronic stress and fear of infection can reduce life satisfaction, limit perceived control over one’s life, and hinder adaptation to demanding professional and personal circumstances. Life satisfaction is a central indicator of subjective well-being and refers to an individual’s global evaluation of their own life, based on the comparison of their actual situation with personal standards and expectations [13,14]. In Diener’s conception, life satisfaction is considered a cognitive component of subjective well-being, distinct from affective components such as positive and negative affect. People with high life satisfaction perceive their lives as consistent with their values and goals, feel competent in managing everyday challenges, and adapt better to changes [15,16]. Conversely, chronic stress, infection risk, work overload, or exposure to patient suffering and death can lead to the accumulation of emotional burdens, reduced sense of control, and ultimately lower overall life evaluation. Such situations may also lead to occupational burnout, which is associated with a negative assessment of job satisfaction [3].

Job satisfaction is defined as a positive evaluation of one’s work experience, encompassing both emotional and cognitive aspects related to the performed tasks, work environment, relationships with supervisors and colleagues, and the sense of personal professional efficacy [17,18]. Literature emphasizes that job satisfaction is an important element of occupational well-being, affecting motivation, efficiency, and engagement in work responsibilities [19]. In the context of the COVID-19 pandemic, healthcare workers’ job satisfaction could have significantly declined. High workload, long working hours, threat of infection, necessity to make difficult medical decisions, and exposure to patient suffering and death increased stress and anxiety levels, negatively affecting the evaluation of one’s work [20,21]. Research has shown that fear of COVID-19 and perceived threat may mediate the relationship between stress and job satisfaction, exacerbating its decline during the pandemic crisis [3,22].

In line with Lazarus and Folkman’s [9] model of stress and coping, the pandemic context, fear of COVID-19, risk perception, and perceived threat can be interpreted as key elements of primary appraisal, which are associated with increased perceived stress. Resources important for coping with the COVID-19 pandemic include, among others: sense of coherence [23], hardiness [24], and self-efficacy [25].

Self-efficacy can be conceptualized as a personal resource in line with resource-based models such as the Job Demands-Resources (JD-R) model and the Conservation of Resources (COR) theory. The JD-R model is a theoretical framework examining employee well-being that divides workplace factors into two main categories: job demands (e.g., pressure, mental strain) and job resources (e.g., supervisory support, autonomy, development opportunities). This model explains that high demands and low resources lead to burnout, while high resources (even with high demands) promote work engagement [2,26,27]

Another increasingly popular approach to understanding stress is Hobfoll’s Conservation of Resources (COR) theory [28], which shares some similarities with relational perspectives on stress. The theory focuses on the principles guiding goal-directed human behavior, emphasizing the central role of resources, with stress emerging when there is a disruption in the balance of resource exchange [29]. According to Hobfoll [28], stress occurs in three primary situations: (1) threat of resource loss, (2) actual loss of resources essential for survival, and (3) lack of expected gain following resource investment. While acknowledging the biological basis of stress, Hobfoll emphasizes the cultural shaping of stress experiences, as society defines the goals individuals pursue. He also proposed four key implications regarding resource dynamics: first, possessing more resources reduces the risk of loss and increases the likelihood of gain, whereas limited resources increase vulnerability to loss and decrease potential gains; second, resource loss tends to trigger further loss cycles; third, resource gain enables further gains, though gain and loss are not equivalent or directly opposite processes; and fourth, individuals with insufficient resources adopt a defensive stance, protecting what they already have [28]. Hobfoll defines resources as objects, conditions, personality traits, and energy reserves that are valued by people, either for survival or to acquire additional resources. The fundamental principle of COR theory is that people strive to obtain, retain, and protect what they value, and stress is particularly pronounced when resources are threatened or lost. Importantly, losses are more salient than gains, meaning the impact of a resource loss is typically stronger than a comparable gain. To prevent losses, compensate for losses, or gain new resources, individuals must invest resources, which can involve direct expenditure or taking risks [28].

Self-efficacy (SE) refers to belief in one’s own abilities and the effectiveness of one’s actions in relation to specific tasks [30]. Bandura [31,32] emphasizes that self-efficacy determines which actions individuals undertake, how much effort they invest, and how long they persist in tasks when facing stressful situations. The level of SE can influence whether an individual will make an effort to cope with a given situation. Self-efficacy is the belief that a person is capable of managing a task or action even in new, unpredictable, difficult, and stressful conditions [33,34].

In stressful situations, such as the pandemic, research has shown that self-efficacy significantly affects individual behavior and well-being. Numerous studies conducted during the COVID-19 pandemic indicate that SE is an important factor related to perceived stress. It was shown that SE correlates with stress levels among nurses [35] and plays a crucial role in coping with COVID-19-related anxiety [36]. Among nurses working in intensive care units, SE was associated with lower perceived stress [37].

Moreover, studies in various groups facing difficult situations have shown that self-efficacy can mediate the relationship between experiencing difficulties and quality of life. Research demonstrated that SE mediated the relationship between experiencing hardships and depression [38] as well as between stress response and long-term suffering after natural disasters [39]. Bandura [30,31], in his social-cognitive theory, emphasizes that in stressful situations, competencies and coping abilities are more important than inherent traits. Self-efficacy is defined as a generalized personal characteristic related to one’s belief in the ability to cope in different life domains [40,41].

SE is considered an important personal resource, playing a key role in coping with challenging situations [42]. The level of SE influences whether an individual will exert effort to meet situational demands [31] and simultaneously determines the choice of coping strategies in stressful conditions. It is also a resource enhancing stress resilience [28,43,44] interacting with concrete, conscious actions undertaken by the individual in response to stress, i.e., coping strategies [9].

Coping strategies are cognitive and behavioral efforts undertaken by an individual in a specific stressful situation. These actions may serve different functions depending on the type of stressor [45]. In the literature, coping strategies are commonly divided into active (considered more effective) and avoidant (less effective). Commonly listed coping strategies include active coping (taking specific actions to solve a problem); planning (developing strategies for action); positive reframing and acceptance (finding a positive aspect or coming to terms with a situation); humor (reducing tension); seeking emotional and instrumental support (contacting others for emotional or practical help); and less adaptive strategies such as avoidance, denial, or emotional surrender [46].

Research indicates that using adaptive strategies, such as active coping, planning, acceptance, seeking support, or humor, is associated with higher well-being, sense of control, and greater job satisfaction [35,36,47]. Conversely, dominance of avoidant and passive strategies may lead to increased stress, helplessness, and reduced life and job satisfaction [9,48].

In light of the above findings, identifying psychological factors that protect healthcare workers from the negative effects of occupational stress in crisis situations, such as the COVID-19 pandemic, becomes particularly important. Previous studies indicate that both self-efficacy and coping strategies may play a crucial role in explaining the relationship between pandemic-related stress and life and job satisfaction.

Although stress can take both positive (eustress) and negative (distress) forms, the present study focuses on distress appraisal, which has been shown to have the strongest links with psychological burden among healthcare workers during the COVID-19 pandemic. Nevertheless, eustress may facilitate motivation, engagement, and active coping strategies. This distinction is conceptually relevant, although it was not captured in our measurement tools.

The integration of perceived stress, coping strategies, and self-efficacy within the mediation framework was grounded in Lazarus and Folkman’s [9] transactional theory of stress and coping, which conceptualizes stress as a result of cognitive appraisal and the subsequent use of coping resources. In this model, perceived stress was treated as the primary antecedent reflecting the initial appraisal of situational demands, whereas coping strategies represented secondary appraisal processes and behavioral responses aimed at regulating stress. Self-efficacy was included as an intrapersonal resource that may influence both the choice and effectiveness of coping responses. Accordingly, the hypothesized mediation model reflected the assumed directional pathway: higher perceived stress influencing satisfaction outcomes indirectly through coping strategies and self-efficacy.

The aim of the present study was to determine the relationships between stress, self-efficacy, coping strategies, and life and job satisfaction, and to identify significant predictors of life and job satisfaction among healthcare workers during the COVID-19 pandemic.

The study hypothesized that:

1. High stress levels would be associated with lower life and job satisfaction, whereas high self-efficacy would be associated with higher levels of both variables.
2. Stress, self-efficacy, and coping strategies would be predictors of life and job satisfaction.
3. Self-efficacy and coping strategies would mediate the relationship between stress and life, and job satisfaction.

## 2. Materials and Methods

### 2.1. Measures

The following research tools were used in the study:

Perceived Stress: The perceived level of stress was measured using the Perceived Stress Scale (PSS-10) by Cohen et al. [49]. This 10-item questionnaire uses a 5-point scale ranging from 0 (never) to 4 (very often). Higher scores indicate greater perceived stress. In the present study, the PSS-10 showed good reliability (Cronbach’s α = 0.84).

Self-Efficacy: General self-efficacy was measured using the Generalized Self-Efficacy Scale (GSES) by Schwarzer and Jerusalem [50]. The questionnaire consists of 10 items rated from 1 (no) to 4 (yes), with higher scores reflecting greater self-efficacy. The scale showed good reliability in the present sample (Cronbach’s α = 0.92).

Coping Strategies: Coping strategies in stressful situations were measured using the Mini-COPE inventory by Carver [51]. The scale consists of 28 items reflecting various coping strategies, grouped into four categories: (I) Active coping (Active Coping, Planning, Positive Reappraisal); (II) Helplessness (Use of Psychoactive Substances, Behavioral Disengagement, Self-Blame); (III) Seeking Support (Emotional Support, Instrumental Support); and (IV) Avoidant Behaviors (Distraction, Denial, Venting of Emotions). Three strategies form independent factors: Turning to Religion, Acceptance, and Sense of Humor. Items are rated on a 4-point scale from 0 (almost never) to 3 (almost always). The scale reliability was good (Cronbach’s α = 0.86).

Job Satisfaction: Job satisfaction was assessed with the Brief Job Satisfaction Scale (BJSS) by Judge et al. [52,53] in the Polish adaptation of Walczak and Derbis [54]. The scale evaluates the cognitive aspect of overall job satisfaction through 5 items (e.g., “I am quite happy with my current job”), ranging from 1 (strongly disagree) to 5 (strongly agree). A higher score on BJSS reflects greater job satisfaction. The presented scale is characterized by good reliability (in this study, Cronbach’s α = 0.84).

Life Satisfaction: Satisfaction with life was assessed using the Satisfaction with Life Scale (SWLS) of Diener et al. [55], specifically the Polish adaptation of Czapiński [56], consisting of 5 items rated from 1 (I definitely disagree) to 7 (I definitely agree). Higher scores indicate greater life satisfaction. In this study, the SWLS demonstrated good reliability (Cronbach’s α = 0.90).

### 2.2. Statistical Analysis

Data preparation, statistical analyses, and part of the tables were performed in Jamovi, Version 2.6 [57]. We used basic descriptive statistics, normality tests, regression diagnostics, and general linear modeling.

### 2.3. Subjects and Procedure

326 medical workers participated in this study (86.81% female, 13.19% male). Most of them work as nurses (32.51%), but there were also doctors (29.75%), midwives (26.69%), and representatives of other medical professions (11.04%), e.g., psychologists, dentists, rehabilitators, and support personnel. The major group was between 41 and 50 (28.83%), lived in small cities (42.33%), and had higher education (80.67%). Respondents most likely reported “mediocre” availability of personal protective equipment, and they usually fear being infected by COVID-19 (92.64%). An a priori Sample Size Calculator [58] for Multiple Regression for anticipated effect size f2 = 0.15, desired statistical power level 0.80, and α = 0.05. suggests a minimum sample size of 113 respondents. The participants’ sociodemographic data are presented in Table 1.

Due to the pandemic context and the dispersed nature of the target group, data collection was carried out online. The study invitation was disseminated online through internal hospital mailing lists, social media groups for healthcare personnel, and professional networks. Participation was voluntary and anonymous, which may have increased the likelihood of response among individuals experiencing higher stress or greater interest in psychological well-being. Due to the recruitment channels, the sample may not be fully representative of all healthcare professions in Poland. Nurses and female healthcare workers were notably overrepresented, which is typical for survey-based research in this occupational group.

Participants were informed about the scientific purpose of the research, the voluntary nature of their participation, and full anonymity of the responses. All participants provided informed consent prior to taking part in the study and were free to withdraw at any stage without any consequences. Completion of the questionnaire was not time-restricted. The study procedure was approved by the Ethics Committee of the University of Opole (approval no. KEBN 15/2021, approved on 26 May 2021) and was conducted in accordance with the ethical standards of the Declaration of Helsinki.

## 3. Results

To verify correlational relations between variables, Spearman’s ρ correlation analysis was performed and presented in Table 2. It appears that life satisfaction correlates positively with work satisfaction (ρ = 0.41; *p* < 0.001), self-efficacy (ρ = 0.43; *p* < 0.001), active coping (ρ = 0.45; *p* < 0.001), social support (ρ = 0.38, *p* < 0.001), and acceptance (ρ = 0.36; *p* < 0.001). There were also negative correlations with perceived stress (ρ = −0.37; *p* < 0.001), helplessness (ρ = −0.33; *p* < 0.001), and avoidance (ρ = −0.11; *p* < 0.05). Work satisfaction correlates positively with life satisfaction (ρ = 0.41; *p* < 0.001), self-efficacy (ρ = 0.33; *p* < 0.001), active coping (ρ = 0.37; *p* < 0.001), and acceptance (ρ = 0.29; *p* < 0.001). There were also negative correlations with perceived stress (ρ = −0.37; *p* < 0.001), helplessness (ρ = −0.33; *p* < 0.001), avoidance (ρ = −0.19; *p* < 0.001), and religious coping (ρ =−0.12; *p* < 0.05).

According to the Shapiro–Wilk test, all variables failed to meet the assumption of normal distribution. None of them exceeded the value of 2 in skewness and kurtosis [59], so the asymmetry is of an acceptable size. The variable with the most asymmetric distribution was work satisfaction (Skewness = −0.70). The biggest kurtosis characterized religious coping (K = −1.29). All the descriptive statistics and Shapiro–Wilk normality test results are presented in Table 3. Due to low reliability, we excluded the humor subscale from further analyses.

In the next step, we checked the assumptions for regression analyses for models with life satisfaction and work satisfaction as dependent variables. We checked autocorrelation with the Durbin–Watson test and multicollinearity via the VIF (variance inflation factor). It turned out that in both models, there were no problems with VIF, and none of the predictors exceeded the value of 5. There was significant autocorrelation in the model with life satisfaction as the dependent variable (DW = 1.73, autocorrelation = 0.133, *p* = 0.006) but not in the model with work satisfaction (DW = 1.93, autocorrelation = 0.03, *p* = 0.540). To minimize the effect of failed regression assumptions, we used the robust SE method (HC3) in regression models and bias-corrected bootstrap (1000 replicates) for mediation models.

The model consists of coping methods, stress, and self-efficacy turned out to significantly predict life satisfaction (R2 = 0.40; F = 26.10; *p* < 0.001). Perceived stress, self-efficacy, and active coping were significant predictors of life satisfaction in this model. The strongest were–social support (β = 0.243; *p* < 0.001) and perceived stress (β = −0.205; *p* < 0.001). Regression coefficients are presented in Table 4.

Coping methods, stress, and self-efficacy form a model that significantly predicts work satisfaction (R2 = 0.27; F = 14.77; *p* < 0.001). Only perceived stress (β = −0.248; *p* < 0.001) and active coping (β = 0.273, *p* < 0.001) turned out to be significant predictors of work satisfaction. Regression coefficients are presented in Table 5.

In the next step, we performed a GLM mediation model analysis to assess whether self-efficacy and coping methods mediate the relationship between stress with life satisfaction. It turned out that there are two significant mediators in this relationship–self-efficacy (β = −0.08; *p* = 0.002; 95% CI [−0.13; −0.02]) and active coping (β = −0.05; *p* = 0.009; 95% CI [−0.08; −0.01]). More detailed results are shown in Table 6. In both mediations, the indirect and direct effects were statistically significant and had the same sign, which means that they are complementary mediations [60].

Afterwards, we examined the model with self-efficacy and coping methods as mediators of the relationship between stress and work satisfaction. Only one mediation was statistically significant–active coping (β = −0.07; *p* = 0.001; 95% CI [−0.09; −0.01]). It has significant indirect and direct effects with the same sign, which means that it is a complementary mediation [60]. More detailed results are shown in Table 7.

## 4. Discussion

The COVID-19 pandemic had a profound impact on healthcare systems worldwide. Due to the risk of infection, increased workload, and ongoing uncertainty about the course of the epidemic, healthcare workers experienced high levels of stress and reduced feelings of safety [3,4]. Therefore, the aim of our study was to determine which individual and psychosocial factors may influence the life and job satisfaction among healthcare workers during the pandemic. Particular attention was paid to the mediating role of self-efficacy and coping strategies in the relationship between stress and life, and job satisfaction.

In our study, we demonstrated that the level of stress experienced by healthcare workers during the COVID-19 pandemic was high. The mean score on the PSS-10 questionnaire in our study was approximately 24, which is clearly higher than the general population score in Poland during the same period (20.6) [61] and above population averages <13 according to Cohen [49], 16.6 according to Juczyński [62]. High stress levels resulted from excessive job demands with limited resources, close contact with COVID-19 patients, the need to adapt to new procedures and protocols, as well as pandemic-related risks of patient death, often in situations of isolation, preventing family presence at the bedside [4]. Correlation analysis showed that both life and job satisfaction were correlated with self-efficacy, stress, and coping strategies. Life and job satisfaction were positively associated with higher self-efficacy, more frequent use of active coping, acceptance, and seeking social support, and negatively associated with perceived stress, helplessness, and avoidance.

These findings are consistent with Lazarus and Folkman’s [9] transactional model of stress and coping, according to which the interpretation of a difficult situation, available resources, and the choice of coping strategies are associated with the level of experienced stress and psychological well-being. Similar relationships were observed in studies conducted among healthcare workers during the COVID-19 pandemic, where effective coping and high self-efficacy were associated with higher job and life satisfaction, as well as lower levels of stress and anxiety [3,4,22,36,37].

Subsequent regression analysis showed that both life satisfaction and job satisfaction during the COVID-19 pandemic were significantly explained by perceived stress, self-efficacy, and coping strategies. The model with life satisfaction as a dependent variable explained 40% of the variance. In this model, significant predictors were perceived stress, self-efficacy, social support, and active coping strategies. The most important predictors were social support and perceived stress.

Stress during the pandemic was associated with overload, feelings of threat, fear of infection, and fear of transmitting the virus to loved ones. Additionally, healthcare workers, particularly nurses, experienced stress related to shortages of personal protective equipment, working in unfamiliar environments, lack of organizational support, conflicts between care duties and protecting their own health, fatigue, and social stigma. All these factors made the pandemic an exceptionally demanding situation for medical personnel, increasing the risk of psychological overload and reducing life satisfaction [4,35,36].

Seeking social support, as one of the active coping strategies, was also a significant predictor of life satisfaction among healthcare workers. Individuals who actively engage with others—both for emotional support (sharing feelings, discussing problems) and instrumental support (asking for practical help)—perceive their lives as more satisfying, exhibit higher well-being, and function better psychologically. During the COVID-19 pandemic, social support was particularly crucial—social isolation, restrictions in interpersonal contact, work overload, or even experiencing stigma made access to people who could listen, advise, or help practically a key element of effective stress coping. In such extreme conditions, social support not only mitigated the effects of stress but also strengthened feelings of control, belonging, and self-worth, consequently increasing life satisfaction, as confirmed in other studies [63,64].

An analogous model with job satisfaction as the dependent variable explained 27% of the variance. In this case, significant predictors were only perceived stress and active coping. These results highlight the dual role of effective coping strategies and stress exposure in shaping employee well-being in high-pressure clinical environments.

First, active coping emerged as a positive predictor of work satisfaction, suggesting that HCWs who engaged in problem-focused and constructive strategies (e.g., planning, taking instrumental action, seeking solutions) were better able to maintain a sense of control and professional fulfillment. This finding aligns with Lazarus and Folkman’s [9] transactional model, which emphasizes coping as a central mechanism that modifies the stress–outcome relationship. It also corresponds with resource-based theories [28], which conceptualize coping efforts as an investment of personal resources that may prevent further losses and generate new gains. In the context of the pandemic, proactive strategies may have helped workers navigate uncertainty, protect key professional resources, and preserve meaning in their work.

In contrast, perceived stress was a significant negative predictor of work satisfaction. As noted by previous research, high stress depletes cognitive, emotional, and social resources, increasing vulnerability to exhaustion, disengagement, and emotional strain [3,4]. These results support the assumption that chronic stress intensifies resource loss cycles [28], which may be especially detrimental in frontline clinical settings where the demands are exceptionally high. This interplay mirrors previous studies conducted during and after the pandemic, showing that healthcare workers’ job satisfaction is shaped by both emotional strain and the availability of psychological resources [65,66,67]. 

Self-efficacy is considered an essential personal resource that plays a key role in coping with difficult situations [42]. The level of self-efficacy can influence whether an individual will exert effort to meet situational demands [31]. It is also a resource that increases stress resilience [32,43,44]. Highly demanding tasks do not paralyze individuals with high self-efficacy because, in stressful situations, they can mobilize, seek new solutions, transform situations, and adapt to adverse conditions [31]. Individuals with strong self-efficacy believe they can cope with difficulties, whereas those with low self-efficacy doubt their abilities, leading to low motivation and reduced readiness to take on challenges [68]. People with high self-efficacy prefer effective coping strategies [69,70] and better control intrusive or distressing thoughts [71].

In the next step, mediation analyses were conducted for both life and job satisfaction. It was shown that self-efficacy and active coping mediate the relationship between stress and life satisfaction. Mediation analysis also showed that active coping mediates the relationship between stress and job satisfaction.

The integration of perceived stress, coping strategies, and self-efficacy within the mediation framework reflects a transactional understanding of stress processes. Perceived stress captures the initial appraisal of the pandemic situation, whereas coping strategies and self-efficacy represent key personal and behavioral resources shaping adaptation. Although alternative conceptual configurations were possible, the tested direction demonstrated a clearer theoretical rationale and stronger empirical fit. This supports the notion that psychological responses to pandemic-related demands unfold through a sequential mechanism in which stress appraisal precedes coping and resource mobilization

Our findings indicate that both active coping and self-efficacy mediated the association between perceived stress and life satisfaction among healthcare workers. This pattern supports the transactional perspective, in which coping efforts and personal beliefs shape the emotional consequences of stress appraisal [9]. Active coping, as a resource-investment strategy, may mitigate the detrimental effects of stress by facilitating a sense of agency and goal-directed problem solving, consistent with Conservation of Resources theory [28]. Similarly, higher self-efficacy appeared to function as a key personal resource that reduces the subjective impact of stress and fosters more adaptive cognitive-emotional regulation, in line with Bandura’s [71] social-cognitive framework.

This mechanism has been confirmed in numerous studies. Peñacoba et al. [37] showed that during the COVID-19 pandemic, self-efficacy mediated the relationship between stress, quality of life, and emotional symptoms among medical staff. Similar relationships were observed among nurses and caregivers, where self-efficacy explained how stress influenced psychological well-being and anxiety levels [72,73]. Kondratowicz et al. [74] also found that self-efficacy mediated the relationship between stress and job satisfaction during remote work. Self-efficacy shapes the interpretation of situational demands, affects coping strategy selection, and helps maintain motivation, making it crucial for life quality. According to Bandura [30,31], individuals with high self-efficacy are more likely to take on challenges, mobilize in difficult situations, and use available resources effectively. During the COVID-19 pandemic, when healthcare workers faced chronic stress, fear of infection, and professional overload, high self-efficacy allowed for more effective coping with daily challenges. Similar to previous studies [35,37], individuals with strong self-efficacy experienced less stress and showed higher motivation, supporting better functioning both professionally and personally.

Active coping emerged as a mediator between stress and job satisfaction, indicating that problem-focused efforts partially explain how stress translates into work-related outcomes. Active coping—defined as deliberate efforts to manage or modify stressors —is consistently associated with better occupational functioning [48]. Employees who engage in planning, problem-solving, and taking concrete steps to handle job demands tend to maintain higher levels of engagement and perceive greater effectiveness in their roles [75,76]. This, in turn, enhances job satisfaction, which is strongly shaped by a sense of control, accomplishment, and successful task completion. Second, active coping appears to be more strongly tied to job satisfaction than other coping strategies because job-related stressors are often controllable or modifiable. Emotion-focused strategies (e.g., avoidance, denial, venting) may reduce distress in the short term but do not change objective working conditions; therefore, they are less predictive of job-related outcomes. Job satisfaction is highly dependent on the match between task demands and the worker’s behavioral engagement—something only active coping provides. Finally, the literature suggests that active coping is particularly protective in high-demand environments such as healthcare. Workers who use proactive strategies maintain a stronger sense of mastery and occupational control, buffering the negative impact of stress on job satisfaction [77,78].Thus, the mediating role of active coping in our model is theoretically coherent and supported by previous empirical evidence.

Our findings show that life and job satisfaction among healthcare workers during the COVID-19 pandemic are significantly associated with perceived stress, self-efficacy, and coping strategies. According to Bandura’s model [31,32] self-efficacy acts as a key personal resource, determining not only direct well-being but also mediating the relationship between stress and life and job satisfaction. Our results indicate that high self-efficacy and adaptive coping strategies can contribute to higher life and job satisfaction. Furthermore, our findings are consistent with previous studies indicating that chronic stress and professional overload may lead to decreased satisfaction and increased risk of burnout [3,4,22]. In this context, coping strategies serve as key adaptive mechanisms—not only reducing negative stress effects but also supporting the mobilization of psychological resources and maintaining a sense of control. Strengthening self-efficacy and promoting adaptive coping strategies may therefore represent an important direction for preventive interventions and support programs for healthcare workers, especially in crisis situations and under chronic stress.

### 4.1. Limitations

Despite the significant findings, this study has some limitations. Its cross-sectional design prevents the establishment of causal relationships between stress, coping strategies, self-efficacy, and satisfaction outcomes. Although mediation analyses may suggest potential pathways, they cannot confirm temporal ordering or directionality. Future longitudinal or experimental designs are needed to validate these mechanisms. All variables were measured using self-report instruments, which may introduce biases such as subjectivity, recall inaccuracies, or social desirability effects. Another limitation worth noting is that the model predicting life satisfaction exhibits residual autocorrelation, indicating that the independence of errors assumption is not fully met, which may lead to lower reliability of results for this model. These factors might affect the precision of associations observed in the model and should be considered when interpreting the findings.

Caution is needed when generalizing the results. The sample was drawn from healthcare workers in Poland, which may limit the applicability of the findings to other cultural settings. Differences in organizational structures, staffing levels, cultural norms, and institutional support may substantially influence stress levels and coping mechanisms among healthcare workers. Countries with more robust occupational health systems, different pandemic management strategies, or greater availability of psychological support may exhibit distinct patterns of associations between stress, coping, self-efficacy, and satisfaction. Future cross-cultural studies would help determine whether the psychological mechanisms identified here hold across healthcare systems with different cultural and organizational characteristics. Additionally, the sample shows a notable gender imbalance. This limits the extent to which findings involving gender can be interpreted, and replication in more balanced samples is needed.

The complementary mediation patterns for all mediation models suggest that although part of the effect is explained through the mediator, the predictor retains a meaningful direct influence on the outcome. This highlights that the mechanism is multifaceted and cannot be reduced to a single mediating process, and some mediators might be omitted in these models.

An additional consideration concerns the potential role of positive stress (eustress). While our study concentrated on distress, eustress may promote adaptive coping, including problem-focused strategies and enhanced self-efficacy. Future research should examine this distinction more explicitly, as differentiating between positive and negative stress responses may help identify protective processes in high-demand clinical environments.

### 4.2. Implications

#### 4.2.1. Theoretical Implications

Our findings contribute to understanding the psychosocial mechanisms influencing well-being among healthcare workers during the COVID-19 pandemic. The study highlights the central role of perceived stress, coping strategies, and self-efficacy in shaping life and job satisfaction. Self-efficacy functions as a personal resource within the framework of resource-based models (JD-R, COR), supporting resilience and adaptive coping. Moreover, the identification of mediation effects clarifies how active coping and self-efficacy interact to influence satisfaction outcomes, offering insights for future theoretical development in occupational and health psychology.

#### 4.2.2. Practical/Clinical Implications

The results underline the importance of promoting active coping strategies and enhancing self-efficacy to support healthcare workers in high-stress clinical environments. Interventions should focus on strengthening personal resources, such as problem-focused coping and positive reappraisal, which are associated with higher job and life satisfaction. Training programs could be implemented to improve self-efficacy in managing stressful situations, while practical stress-management techniques can enhance the use of effective coping strategies. Recognizing both positive and negative forms of stress (eustress and distress) can guide the design of interventions that foster engagement and motivation, while mitigating harmful stress effects.

#### 4.2.3. Directions for Future Research

Further studies should explore the generalizability of these findings across different cultural, occupational, and organizational contexts, considering variations in healthcare systems. Comparing different occupational groups and considering cultural and organizational factors influencing social support perception could further enrich our understanding of well-being in healthcare workers. Longitudinal designs could provide more robust evidence on causal relationships between stress, coping, and self-efficacy. Additionally, future research could investigate the role of personality traits, hope, and gratitude in moderating affective responses and coping efficiency, as well as the dynamics of coping strategies under repeated or prolonged stress. These insights could inform targeted interventions tailored to specific subgroups of healthcare workers.

## 5. Conclusions

In summary, perceived stress, self-efficacy, and adaptive coping strategies such as acceptance and seeking social support are key predictors of life and job satisfaction among healthcare workers during the COVID-19 pandemic. These findings suggest that interventions aimed at strengthening self-efficacy and promoting adaptive coping could enhance well-being and resilience in healthcare workers facing high-stress conditions.

## Figures and Tables

**Table 1:** Characteristics of the studied sample (N = 326).

	N (%)
Gender	
Woman	283 (86.81)
Man	43 (13.19)
Age	
30 and below	90 (27.61)
31–40	65 (19.94)
41–50	94 (28.83)
51–60	66 (20.24)
More than 60	3 (2.83)
Profession	
Doctor	91 (29.75)
Nurse	106 (32.51)
Midwife	87 (26.69)
Other	36 (11.04)
Residence	
Village	67 (20.55)
Small city	138 (42.33)
Provincial city	121 (37.12)
Education	
Higher ^1^	263 (80.67)
Secondary	63 (19.32)

^1^ higher education is defined here as holding either a bachelor’s or a master’s degree.

**Table 2:** Spearman’s ρ coefficient, Cronbach’s α, and McDonald’s ω for psychological variables from the study.

	1	2	3	4	5	6	7	8	9	10	11
1. Life satisfaction	—										
2. Work satisfaction	0.411 ***	—									
3. Perceived stress	−0.368 ***	−0.373 ***	—								
4. Self-efficacy	0.425 ***	0.325 ***	−0.406 ***	—							
5. COPE active coping	0.448 ***	0.370 ***	−0.235 ***	0.420 ***	—						
6. COPE helplessness	−0.329 ***	−0.342 ***	0.494 ***	−0.427 ***	−0.285 ***	—					
7. COPE social support	0.383 ***	0.100	0.026	0.126 *	0.336 ***	−0.025	—				
8. COPE avoidance	−0.114 *	−0.193 ***	0.376 ***	−0.170 **	−0.009	0.521 ***	0.172 **	—			
9. COPE religious coping	0.083	−0.119 *	0.054	−0.148 **	0.024	0.131 *	0.203 ***	0.304 ***	—		
10. COPE acceptance	0.359 ***	0.287 ***	−0.363 ***	0.430 ***	0.506 ***	−0.321 ***	0.198 ***	−0.187 ***	−0.020	—	
11. COPE humor	0.133 *	0.083	−0.164 **	0.261 ***	0.182 ***	0.041	0.101	0.142 *	−0.114 *	0.283 ***	—
Cronbach’s α	0.90	0.88	0.84	0.92	0.75	0.78	0.89	0.72	0.91	0.66	0.13
McDonald’s ω	0.90	0.89	0.85	0.92	0.76	0.78	0.89	0.73	0.91	0.66	0.25

Note. * *p* < 0.05; ** *p* < 0.01; *** *p* < 0.001.

**Table 3:** Descriptive statistics with Shapiro–Wilk’s normality tests.

	Mean	Median	SD	Skewness	Kurtosis	W	*p*
Life satisfaction	24.03	24.00	5.54	−0.55	0.42	0.98	<0.001
Work satisfaction	18.18	19.50	4.66	−0.70	−0.28	0.94	<0.001
Perceived stress	23.92	24.00	6.73	−0.14	−0.75	0.98	<0.001
Self-efficacy	28.81	29.00	4.93	−0.37	1.08	0.96	<0.001
COPE active coping	2.08	2.00	0.50	−0.23	−0.05	0.98	<0.001
COPE helplessness	0.97	0.83	0.64	0.60	−0.42	0.95	<0.001
COPE social support	1.99	2.00	0.74	−0.60	0.05	0.94	<0.001
COPE avoidance	1.52	1.50	0.63	0.36	−0.01	0.98	<0.001
COPE religious coping	1.25	1.00	1.09	0.31	−1.29	0.87	<0.001
COPE acceptance	2.02	2.00	0.63	−0.41	0.09	0.92	<0.001
COPE humor	0.95	1.00	0.64	0.40	−0.22	0.93	<0.001

**Table 4:** Regression coefficients for the model predicting life satisfaction.

						β 95% Confidence Intervals	
Predictor	B	SE	t	*p*	β	Lower	Upper
Intercept	24.03	0.24	99.59	<0.001		−0.086	0.086
Perceived stress	−0.17	0.04	−4.03	<0.001	−0.205	−0.312	−0.098
Self-efficacy	0.21	0.07	2.89	0.004	0.183	0.070	0.297
COPE active coping	1.92	0.73	2.63	0.009	0.175	0.063	0.287
COPE helplessness	−1.02	0.53	−1.95	0.052	−0.119	−0.243	0.005
COPE social support	1.83	0.41	4.46	<0.001	0.243	0.149	0.338
COPE avoidance	0.21	0.47	0.45	0.654	0.024	−0.094	0.142
COPE religious coping	0.34	0.24	1.41	0.159	0.067	−0.027	0.162
COPE acceptance	0.53	0.45	1.18	0.240	0.061	−0.047	0.169
R2 = 0.40; F (9, 316) = 26.10; *p* < 0.001							

Note. Inferential tests and *p*-values are based on robust estimation.

**Table 5:** Regression coefficients for the model predicting work satisfaction.

						β 95% Confidence Intervals	
Predictor	B	SE	t	*p*	β	Lower	Upper
Intercept	18.18	0.23	80.05	<0.001		−0.094	0.094
Perceived stress	−0.17	0.04	−4.23	<0.001	−0.248	−0.365	−0.130
Self-efficacy	0.08	0.07	1.11	0.250	0.080	−0.044	0.205
COPE active coping	2.52	0.72	3.50	<0.001	0.273	0.149	0.396
COPE helplessness	−0.46	0.54	−0.85	0.385	−0.063	−0.199	0.073
COPE social support	−0.12	0.32	−0.37	0.700	−0.019	−0.123	0.085
COPE avoidance	0.11	0.49	0.21	0.828	0.014	−0.116	0.144
COPE religious coping	−0.30	0.23	−1.34	0.173	−0.071	−0.174	0.033
COPE acceptance	0.21	0.52	0.40	0.680	0.028	−0.09	0.146
R2 = 0.27; F (9, 316) = 14.77; *p* < 0.001							

Note. Inferential tests and *p*-values are based on robust estimation.

**Table 6:** Indirect and total effects for the model explaining life satisfaction.

				95% C.I.				
**Type**	Effect	Estimate	SE	Lower	Upper	β	z	*p*
Indirect	Perceived stress ⇒ Self-efficacy ⇒ Life satisfaction	−0.07	0.022	−0.119	−0.02	−0.081	−3.03	0.002
	Perceived stress ⇒ Active coping ⇒ Life satisfaction	−0.04	0.014	−0.078	−0.009	−0.046	−2.63	0.009
	Perceived stress ⇒ Helplessness ⇒ Life satisfaction	−0.05	0.026	−0.098	0.001	−0.060	−1.88	0.060
	Perceived stress ⇒ Social support ⇒ Life satisfaction	0.01	0.011	−0.014	0.030	0.008	0.59	0.554
	Perceived stress ⇒ Avoidance ⇒ Life satisfaction	0.01	0.019	−0.027	0.042	0.009	0.41	0.685
	Perceived stress ⇒ Religious coping ⇒ Life satisfaction	0.01	0.004	−0.003	0.02	0.004	0.86	0.389
	Perceived stress ⇒ Acceptance ⇒ Life satisfaction	−0.02	0.017	−0.053	0.010	−0.023	−1.12	0.265
Component	Perceived stress ⇒ Self-efficacy	−0.32	0.036	−0.403	−0.247	−0.441	−8.88	<0.001
	Self-efficacy ⇒ Life satisfaction	0.21	0.064	0.061	0.348	0.183	3.23	0.001
	Perceived stress ⇒ Active coping	−0.02	0.004	−0.028	−0.011	−0.262	−4.91	<0.001
	Active coping ⇒ Life satisfaction	1.92	0.618	0.317	3.369	0.175	3.11	0.002
	Perceived stress ⇒ Helplessness	0.05	0.005	0.04	0.057	0.507	10.63	<0.001
	Helplessness ⇒ Life satisfaction	−1.02	0.536	−2.008	0.033	−0.119	−1.91	0.056
	Perceived stress ⇒ Social support	0.01	0.006	−0.008	0.014	0.033	0.60	0.551
	Social support ⇒ Life satisfaction	1.83	0.355	1.062	2.623	0.243	5.14	<0.001
	Perceived stress ⇒ Avoidance	0.04	0.005	0.027	0.045	0.382	7.47	<0.001
	Avoidance ⇒ Life satisfaction	0.21	0.524	−0.793	1.15	0.024	0.41	0.685
	Perceived stress ⇒ Religious coping	0.01	0.009	−0.011	0.025	0.060	1.08	0.280
	Religious coping ⇒ Life satisfaction	0.34	0.24	−0.154	0.844	0.067	1.43	0.154
	Perceived stress ⇒ Acceptance	−0.04	0.005	−0.045	−0.026	−0.379	−7.39	<0.001
	Acceptance ⇒ Life satisfaction	0.53	0.473	−0.278	1.488	0.061	1.13	0.259
Direct	Perceived stress ⇒ Life satisfaction	−0.17	0.044	−0.249	−0.085	−0.205	−3.82	<0.001
Total	Perceived stress ⇒ Life satisfaction	−0.32	0.042	−0.409	−0.240	−0.394	−7.72	<0.001

Note. Betas are completely standardized effect sizes. Confidence intervals were computed using a bias-corrected bootstrap (1000 resamples).

**Table 7:** Indirect and total effects for the model explaining work satisfaction.

				95% C.I.				
Type	Effect	Estimate	SE	Lower	Upper	β	z	*p*
Indirect	Perceived stress ⇒ Self-efficacy ⇒ Work satisfaction	−0.03	0.019	−0.075	0.013	−0.035	−1.27	0.203
	Perceived stress ⇒ Active coping ⇒ Work satisfaction	−0.05	0.015	−0.089	−0.021	−0.072	−3.28	0.001
	Perceived stress ⇒ Helplessness ⇒ Work satisfaction	−0.02	0.024	−0.07	0.034	−0.032	−0.92	0.360
	Perceived stress ⇒ Social support ⇒ Work satisfaction	0.01	0.001	−0.007	0.002	−0.001	−0.31	0.755
	Perceived stress ⇒ Avoidance ⇒ Work satisfaction	0.01	0.017	−0.028	0.042	0.005	0.22	0.829
	Perceived stress ⇒ Religious coping ⇒ Work satisfaction	−0.01	0.003	−0.017	0.002	−0.004	−0.85	0.397
	Perceived stress ⇒ Acceptance ⇒ Work satisfaction	−0.01	0.016	−0.044	0.03	−0.011	−0.47	0.637
Component	Perceived stress ⇒ Self-efficacy	−0.32	0.036	−0.4	−0.244	−0.441	−8.88	<0.001
	Self-efficacy ⇒ Work satisfaction	0.08	0.059	−0.047	0.213	0.080	1.29	0.198
	Perceived stress ⇒ Active coping	−0.02	0.004	−0.027	−0.011	−0.262	−4.91	<0.001
	Active coping ⇒ Work satisfaction	2.52	0.572	1.051	3.77	0.273	4.41	<0.001
	Perceived stress ⇒ Helplessness	0.05	0.005	0.04	0.057	0.507	10.63	<0.001
	Helplessness ⇒ Work satisfaction	−0.46	0.496	−1.413	0.718	−0.063	−0.92	0.358
	Perceived stress ⇒ Social support	0.01	0.006	−0.008	0.014	0.033	0.60	0.551
	Social support ⇒ Work satisfaction	−0.12	0.329	−0.672	0.579	−0.019	−0.37	0.713
	Perceived stress ⇒ Avoidance	0.04	0.005	0.027	0.044	0.382	7.47	<0.001
	Avoidance ⇒ Work satisfaction	0.11	0.484	−0.811	1.186	0.014	0.22	0.829
	Perceived stress ⇒ Religious coping	0.01	0.009	−0.009	0.028	0.060	1.08	0.280
	Religious coping ⇒ Work satisfaction	−0.30	0.222	−0.725	0.116	−0.071	−1.36	0.172
	Perceived stress ⇒ Acceptance	−0.04	0.005	−0.045	−0.026	−0.379	−7.39	<0.001
	Acceptance ⇒ Work satisfaction	0.21	0.437	−0.846	1.214	0.028	0.47	0.636
Direct	Perceived stress ⇒ Work satisfaction	−0.17	0.041	−0.254	−0.092	−0.248	−4.20	<0.001
Total	Perceived stress ⇒ Work satisfaction	−0.28	0.035	−0.338	−0.209	−0.397	−7.79	<0.001

Note. Betas are completely standardized effect sizes. Confidence intervals were computed using a bias-corrected bootstrap (1000 resamples).

## Data Availability

The data can be made available from the corresponding author upon reasonable request.
